# Clinical value of microRNA-378a-3p in sepsis and its role in sepsis-induced inflammation and cardiac dysfunction

**DOI:** 10.1080/21655979.2021.1985339

**Published:** 2021-10-21

**Authors:** Qing Wang, Kuo Liu, Changming Jin

**Affiliations:** Department Of Emergency, Emergency General Hospital, Beijing, China

**Keywords:** Sepsis, miR-378a-3p, diagnosis, biomarker

## Abstract

This study explored the clinical meaning of miR-378a-3p in sepsis and its influence on sepsis-induced inflammation and cardiac dysfunction. Serum levels of miR-378a-3p were detected by quantitative Real-Time Polymerase Chain Reaction (qRT-PCR). The Receiver Operating Characteristic (ROC) curve was used to evaluate its diagnostic value. The effects of miR-378a-3p on inflammation and cardiac function were evaluated by monitoring left ventricular systolic pressure (LVSP), left ventricular and end-diastolic pressure (LVEDP), maximum rate of change in left ventricular pressure (± dp/dt_max_) and detecting the levels of troponin I (cTnI), creatine kinase isoenzyme MB (CK-MB), tumor necrosis factor-a (TNF-a), interleukin-6 (IL-6), and interleukin-1β (IL-1β) via enzyme linked immunosorbent assay (ELISA). Serum miR-378a-3p was increased in sepsis patients and rat model. ROC curve indicated that miR-378a-3p might have diagnostic significance for sepsis miR-378a-3p antagomir improved the cardiac function by upregulating the levels of LVSP and ± dp/dt_max_, and decreasing the levels of LVEDP, cTnI and CK-MB in rat model. miR-378a-3p antagomir also significantly alleviated the inflammatory responseby down-regulating the expression of TNF-a, IL-6, and IL-1β. Besides, logistics regression analysis illustrated that miR-378a-3p was an independent influencing factor for the onset of cardiac dysfunction in sepsis. miR-378a-3p has the potential as a diagnostic biomarker for sepsis and decreasing the level of miR-378a-3p had the ability to ameliorate cardiac dysfunction and inflammatory response caused by sepsis.

## Introduction

Sepsis is an unusual systemic reaction caused by the dysregulation of the immune inflammatory response to infection [1]. Sepsis, a common complication after severe trauma, burns and surgery, can lead to septic shock and multiple organ dysfunction, and has become one of the important causes of death in critically ill patients [2]. Clinically, sepsis patients are often treated with antimicrobial, mechanical ventilation, electrolyte balance maintenance and drug therapy, but the prognosis is still not satisfactory [3]. The organ dysfunction caused by sepsis is the primary factor for its high mortality rate [4]. It was reported that in sepsis, the heart is one of the most vulnerable organs, with nearly 50% of patients eventually developing cardiac dysfunction [5,6]. Studies have shown that about 50% of patients with sepsis have severe cardiac dysfunction, and the mortality rate is as high as nearly 70%. However, at present, the mechanism of sepsis mediated cardiac dysfunction is not very clear, which may involve multiple factors such as inflammatory response, microcirculation disturbance, activation of endothelial cells, and abnormal distribution of coronary blood flow [7]. Microbial culture was once considered as the gold standard for the diagnosis of sepsis, but it is not conducive to the early diagnosis of sepsis due to its time-consuming and low accuracy [8]. Therefore, searching for a new biomarker for sepsis, such an acute and serious disease, is undoubtedly a breakthrough new approach for diagnosis of sepsis.

MicroRNA (miRNA) is a type of endogenous non-coding RNA widely present in eukaryotic cells. It was highly conserved and was found to be involved in regulating the growth, development, differentiation, and apoptosis of organisms [9]. It has been reported that miR-223 was abnormally expressed in sepsis and rheumatoid arthritis [10]. The expression of miR-223 in the cardiomyocytes of mice with severe sepsis is extremely low, and the loss of miR-223 made the cardiac insufficiency of mice more serious [11]. Stefano et al. reported that miR-378a-3p, as a circulating inflammation-related microRNA, was significantly positively correlated with SOFA score in systemic inflammatory response syndrome (SIRS) [12]. Sepsis shares many clinical features with SIRS, such as leukocytosis and tachycardia [13]. So far, there has been no research on whether miR-378a-3p participated in the regulation of sepsis, or how the regulation of miR-378a-3p is carried out and what are the molecular mechanisms.

Considering the role of miR-378a-3p in inflammation, we hypothesized that miR-378a-3p plays a regulatory role in sepsis. We evaluated the level of miR-378a-3p in serum of patients with sepsis, and studied the effects of miR-378a-3p on cardiac function and inflammation in sepsis rats. This study showed abnormal expression of miR-378a-3p in sepsis and miR-378a-3p plays a role in cardiac dysfunction in rat model, which provides an experimental basis for exploring the diagnosis of sepsis and its influence on cardiac function and inflammation.

## Materials and methods

### Subject information and sample collection

A total of 80 sepsis patients admitted to the intensive care unit (ICU) of Emergency General Hospital from November 2017 to June 2020 and 72 healthy people who underwent physical examination in this hospital during the same period were selected as the research subjects. The diagnostic criteria for sepsis follow the International Diagnostic Criteria for Sepsis 3.0 published by SCCM/ESICM in 2016. Sepsis can be determined if the following two conditions are met simultaneously, 1) suspected infection or infection; 2) sequential organ failure assessment (SOFA) score ≥ 2 [14]. All subjects were excluded as follows: (1) people with congenital immune deficiency, (2) people who carried immunodeficiency virus, (3) people with severe hepatic or renal dysfunction, or with previous chronic cardiac insufficiency, acute myocardial infarction or atrial fibrillation, (4) blood system disease, (5) malignant tumor, (6) people who had taken immunosuppressive drugs within 3 months. Blood samples were collected from sepsis patients admitted to the ICU for the first time within 24 hours, and was used for the detection of serum creatinine (Scr), Albumin, white blood cell (WBC), C-reactive protein (CRP) and procalcitonin (PCT) which were recorded by researchist. The patients of acute physiological and chronic health (APACHE-II) and SOFA were scored. General clinical indicators of all subjects, such as age, gender, and body mass index (BMI) were collected and recorded for analysis. All sepsis patients were monitored and recorded during the treatment. According to the diagnostic criteria of cardiac dysfunction defined by the Heart Review and Evaluation Committee (CREC), sepsis patients were divided into patients with cardiac dysfunction group and patients without cardiac dysfunction group. Criteria for cardiac dysfunction are as follows: 1) Cardiomyopathy characterized by an overall decline in LVEF; 2) Symptoms or signs of heart failure; 3) LVEF decreases by at least 5% to below 55% with signs or symptoms of heart failure; 4) LVEF decreases by at least 10% to below 55% without signs or symptoms of heart failure [15].

Based on 5% of false positive error rate (two-sided, α = 0.05), power = 90%, β = 0.1, and dropout rate = 20%, PASS 11.0 software (NCSS, Kaysville, UT, USA) was used to estimate the sample size, and at least 70 subjects in each group should be recruited as the minimum sample size limit. Therefore, the sample size of this study meets the requirements.

This study was carried out after obtaining the permission of the Ethics Committee of Emergency General Hospital. Subjects and their family members were informed of the study and signed the written informed consent.

### Establishment of sepsis rat model

The animal experiments involved in our study were implemented in the light of the guidelines for animal care and use promulgated by the ethics committee of Emergency General Hospital. 40 adult male Sprague-Dawley (SD) rats with a body weight of 250 g-300 g were purchased from Shanghai Animal Center as modeling objects. All the rats were placed at 23 ± 1°C with 50% humidity, and alternating light and darkness. In this study, cecum ligation and puncture (CLP) are used to replicate the rat sepsis model based on the previously published methods [16]. Briefly, SD rats were anesthetized with sodium pentobarbital (50 mg/kg), and a 1.5 cm of surgical incision was made in the middle of the anterior abdomen. Next, the skin was incised, and the cecum was lifted out. The cecum was ligated 1.5 cm away from the cecum with line 1. At the central part of the distal cecal ligation, perforation was performed with the sterile needle 18 for 3 times. Finally, the cecum was placed back into the abdominal cavity and the abdominal incisions were sutured in layers. Control group received no cecal ligation and perforation, and other operations were the same as those in the CLP group.

### Animal grouping and treatment

SD rats were randomly divided into control group, CLP group, miR-378a-3p negative control group (miR-378a-3p NC group) and miR-378a-3p antagomir group, with 10 rats in each group, and all the other groups received CLP surgery except for control group. SD rats in control group and CLP group were injected with normal saline through caudal vein before operation. In miR-378a-3p NC group and miR-378a-3p antagomir group, 10 μg of corresponding drugs were injected into rats via caudal vein before operation. Both miR-378a-3p NC and miR-378a-3p antagomir used in this study were synthesized and provided by Genepharma. Co., Ltd. The sequences of miRNAs used are described below, miR-378a-3p NC: 5ʹ-UGCAUGUUAUGCCUACG-3ʹ, miR-378a-3p antagomir: 5ʹ-UUCUGACUCCAAGUCCA-3ʹ.

### Monitoring of hemodynamic indexes

At 48 hours following surgery, the changes of hemodynamic parameters in the above 4 groups of SD rats were detected to evaluate their cardiac function. MFLab 3.01 software in FDP-1 HRV & BRS system (Shanghai, China) was used for indicator monitoring [17], including left ventricular systolic pressure (LVSP), left ventricular and end-diastolic pressure (LVEDP) and maximum rate of change in left ventricular pressure (± dp/dt_max_).

### Enzyme linked immunosorbent assay (ELISA)

After the completion of the monitoring of hemodynamic indicators, the expression levels of inflammatory cytokines, such as tumor necrosis factor-α (TNF-α), interleukin-6 (IL-6), and interleukin-1β (IL-1β), as well as myocardial injury markers, troponin I (cTnI), creatine kinase isoenzyme MB (CK-MB) in serum of rats were detected to evaluate the cardiac function and inflammation levels in each group after operation. The serum expressions of cTnI, CK-MB, TNF-α, IL-6, and IL-1β were measured by ELISA kit (Dakewe Biotech, Beijing, China) according to the manufacturer’s protocol.

### Quantitative real-time polymerase chain reaction (qRT-PCR)

Total RNAs in human serum, including 80 sepsis patients and 72 healthy controls, as well as rat serum were extracted and reverse transcribed into cDNA by PrimeScript™ RT reagent Kit (TaKaRa, Dalian, China) under the specified conditions. ABI PRISM 7000 (Applied biomes, USA) plus miScript SYBR® Green PCR kit (Qiagen, Hilden, Germany) was performed for PCR analysis. U6 was regarded as an internal reference, and 2^−ΔΔCt^ method was used to calculate the expression level of miR-378a-3p.

### Data analysis

GraphPad Prism 7.0 and SPSS 19.0 were carried out for data analysis. All experiments were repeated three times and corresponding data were presented as mean ± standard deviation (SD). Student *t* test was used for two groups’ comparison, and one-way ANOVA followed with Tukey post-hoc test was served as multi-groups’ comparison. The categorical variables were analyzed by chi-square test. Receiver operating characteristic curve assessed the diagnostic value of miR-378a-3p in sepsis. The correlation between miR-378a-3p and clinical indicators was evaluated using Pearson’s analysis. Logistic regression analysis was adopted to evaluate the effects of different factors on sepsis. *P* < 0.05 represents a significant difference.

## Results

### Demographic and clinical characteristics of the subjects

A total of 152 participants recruited in this study, including 80 sepsis patients and 72 healthy controls. The baseline data of all subjects are presented in Table 1. We could clearly see from the table that there was no significant difference between the healthy control group and the sepsis patient group in age, gender, and body mass index (BMI) (*P* > 0.05). It was worth noting that the differences in serum creatinine (Scr), albumin, white blood cell (WBC), C-reactive protein (CRP) and procalcitonin (PCT) between the two groups were statistically significant (*P* < 0.001), and the elevation of WBC, CRP and PCT indicated that sepsis patients have accompanied the occurrence of inflammatory response. Furthermore, the APACHE II and SOFA scores of the sepsis patients were 12.53 ± 3.34 and 5.24 ± 1.35, respectively. A higher SOFA score also indicated the severity of organ dysfunction in sepsis patients.

**Table 1.** Comparison of the clinical charateristics between the two groups of study objects

Clinical variables	Health individuals (n = 72)	Sepsis patients (n = 80)	*P*
Age (year)	53.44 ± 11.50	54.61 ± 11.29	0.529
Gender (male/female)	28/44	39/41	0.221
BMI (kg/m^2^)	21.79 ± 2.96	22.59 ± 3.21	0.114
Scr (mg/dL)	1.12 ± 0.26	1.57 ± 0.41	***
Albumin (g/L)	41.25 ± 5.43	26.79 ± 4.31	***
WBC (×10^9^/L)	7.14 ± 1.53	16.73 ± 5.02	***
CRP (mg/L)	5.55 ± 2.98	79.64 ± 15.97	***
PCT (ng/mL)	0.05 ± 0.02	11.41 ± 3.78	***
APACHE II score	-	12.53 ± 3.34	-
SOFA score	-	5.24 ± 1.35	-

Note: BMI, body mass index; Scr, serum creatinine; WBC, white blood cell; CRP, C-reactive protein; PCT, procalcitonin; APACHE, acute physiology and chronic health evaluation; SOFA, sequential organ failure assessment; ***, *P* < 0.001.

### Expression level of miR-378a-3p in sepsis patients

**Figure 1.**
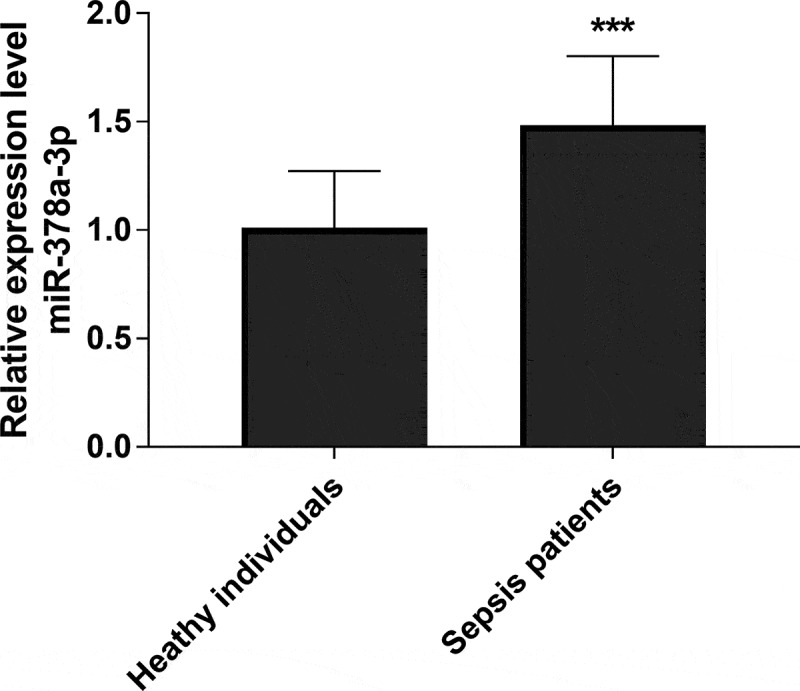
qRT-PCR analysis of serum expression level of miR-378a-3p in sepsis patients and healthy controls

To detect the expression of miR-378a-3p in serum of sepsis patients and sepsis rat model, qRT-PCR analysis was performed. As exhibited in Figure 1, qRT-PCR analysis demonstrated that the serum level of miR-378a-3p in sepsis patients was upregulated significantly in comparison to healthy controls, which presented a 1.48-fold increase (*P* < 0.001). Therefore, we hypothesized that miR-378a-3p may play a momentous role in sepsis through this experimental result.

*** *P* < 0.001.

### ROC analysis

**Figure 2.**
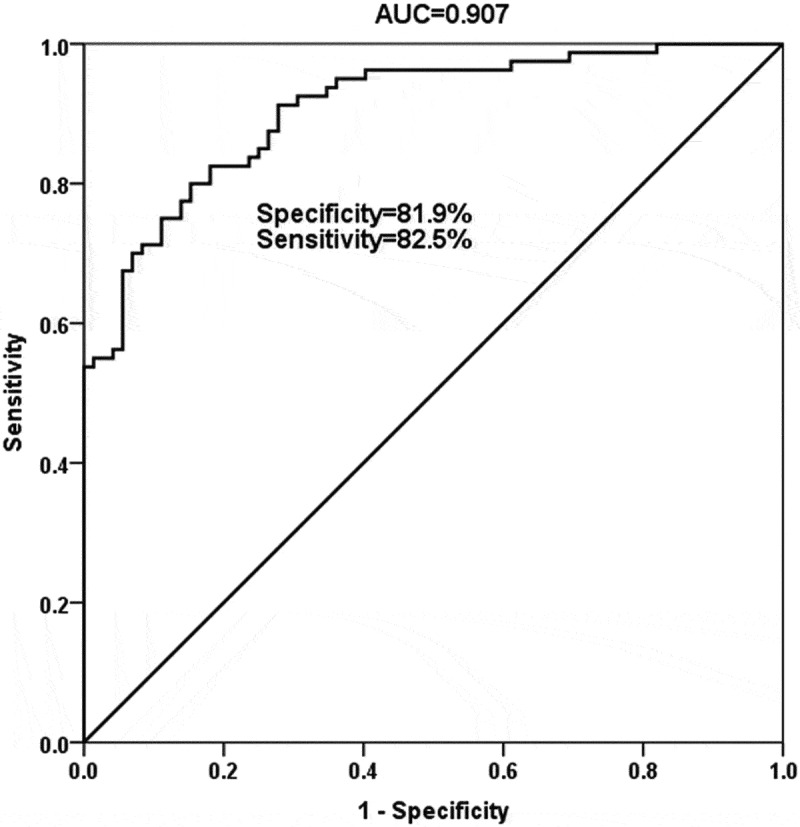
The receiver operating characteristic curve was established to evaluate the diagnostic value of miR-378a-3p in sepsis patients

Considering the abnormal expression of miR-378a-3p in sepsis, we evaluated the clinical diagnostic value of miR-378a-3p in sepsis by ROC curve. The ROC curve confirmed the diagnostic meaning of miR-378a-3p in sepsis. In Figure 2, the curve had high AUC, sensitivity, and specificity values, with the values of 0.907, 82.5% and 81.9%, respectively. Results illustrated that miR-378a-3p had the ability to distinguish sepsis patients from healthy people.

### Pearson correlation coefficient and Logistic regression analysis

Based on the current study, we evaluated the correlation between miR-378a-3p and clinical indicators of patients and the relationship between miR-378a-3p and the occurrence of cardiac insufficiency through Pearson correlation coefficient and Logistic regression analysis, respectively. Pearson correlation coefficient was used to estimate the correlation between miR-378a-3p and clinicopathological characteristics of sepsis patients. It was observed that a significant positive correlation in the expression level of miR-378a-3p with WBC, PCT, APACHE II score and SOFA score (Table 2, *P* < 0.05), suggesting that the level of miR-378a-3p was positively associated with the severity of sepsis. Besides, sepsis patients were grouped into cardiac dysfunction group (n = 42) and normal group (n = 38) according to the results of cardiac function monitoring. Logistic regression analysis was used to evaluate the relationship between miR-378a-3p and the occurrence of cardiac dysfunction. The results shown in Table 3 revealed that miR-378a-3p was an independent influencing factor for the occurrence of cardiac dysfunction (OR = 3.000, 95% CI = 1.077–8.356, *P* = 0.036) in sepsis patients.

**Table 2.** The relation of miR-378a-3p expression with the clinical variables

Parameters	Correlation (r)	*P*
Scr (mg/dL)	0.040	0.725
Albumin (g/L)	0.019	0.868
WBC (×10^9^/L)	0.412	<0.001
CRP (mg/L)	0.194	0.084
PCT (ng/mL)	0.319	0.004
APACHE II score	0.432	<0.001
SOFA score	0.698	<0.001

Note: BMI, body mass index; Scr, serum creatinine; WBC, white blood cell; CRP, C-reactive protein; PCT, procalcitonin; APACHE, acute physiology and chronic health evaluation; SOFA, sequential organ failure assessment.

**Table 3.** Association of different variables with the occurrence of cardiac dysfunction

Variables	OR	95% CI	*P* value
MiR-378a-3p	3.000	1.077–8.356	0.036
Age (year)	1.091	0.402–2.958	0.864
Gender (male/female)	1.642	0.603–4.475	0.332
BMI (kg/m^2^)	1.651	0.628–4.344	0.310
Scr (mg/dL)	1.484	0.516–4.270	0.464
Albumin (g/L)	1.513	0.525–4.356	0.443
WBC (×10^9^/L)	1.169	0.427–3.203	0.761
CRP (mg/L)	1.148	0.397–3.324	0.798
PCT (ng/mL)	1.917	0.665–5.841	0.221
APACHE II score	1.506	0.539–4.207	0.435
SOFA score	2.853	0.915–8.901	0.071

Abbreviations: BMI, body mass index; Scr, serum creatinine; WBC, white blood cell; CRP, C-reactive protein; PCT, procalcitonin; APACHE, acute physiology and chronic health evaluation; SOFA, sequential organ failure assessment

### Effects of miR-378a-3p on cardiac function in sepsis rat model

**Figure 3.**
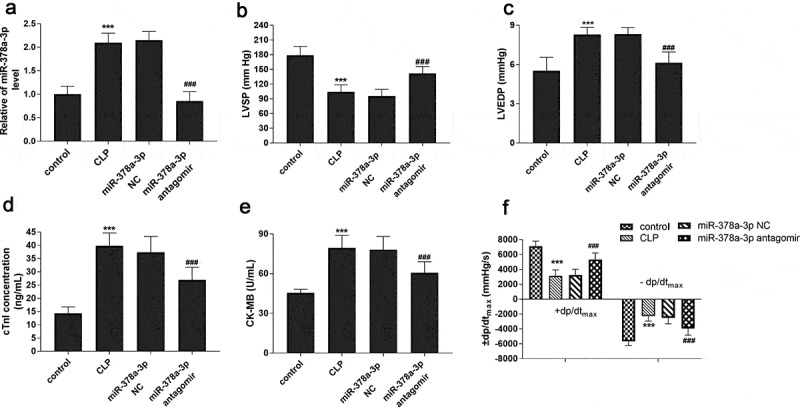
Effects of miR-378a-3p on cardiac function in sepsis rat models. (a) Serum miR-378a-3p expression in different groups of rat models. Changes of (b) LVSP, (c) LVEDP, (d) cTnI, (e) CK-MB and (f) ± dp/dt_max_ in experimental groups

Sepsis rat model was established to explore the effect of miR-378a-3p on cardiac function in septic rats. Figure 3a reveals such a phenomenon, the expression of miR-378a-3p in the serum of sepsis rats was highly expressed, which was consistent with the result in the serum of sepsis patients (*P* < 0.001). Moreover, the LVSP and +dp/dt_max_ of the CLP model group showed a significant decreasing trend, while LVEDP, -dp/dt_max_, cTnI and CK-MB exhibited a relative upward trend (Figure 3b-f, *P* < 0.001), indicating that the cardiac function of sepsis rat model had been disturbed. Meanwhile, another interesting result was observed. That is, the downregulation or upregulation of these indicators of cardiac dysfunction could be successful turned around by the administration of miR-378a-3p antagomir. Based on this, we considered that upregulated miR-378a-3p may exacerbate sepsis by affecting cardiac function.

*** *P* < 0.001 vs. control group, **^###^** *P* < 0.001 vs. CLP group.

### Effects of miR-378a-3p on inflammatory response in sepsis rat model

**Figure 4.**
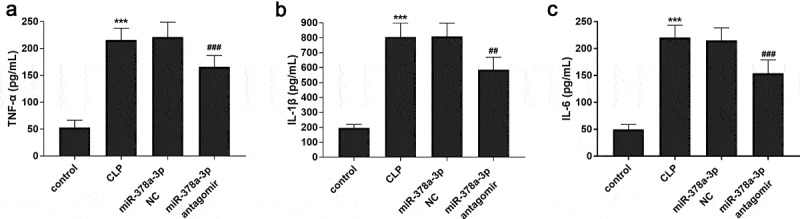
Effects of miR-378a-3p on inflammatory response in sepsis rat models. Changes of (a) TNF-α, (b) IL-1β and (c) IL-6 in the serum of sepsis rats

To further explore whether miR-378a-3p is involved in regulating inflammatory response, the inflammatory response of the sepsis rat model was evaluated by the levels of TNF-α, IL-1β, IL-6, and the results are shown in Figure 4a-c. It was found that the expressions of TNF-α, IL-1β, IL-6 in CLP group were significantly increased (*P* < 0.001), suggesting that animal’s inflammatory response was activated by sepsis. Besides, the injection of miR-378a-3p antagomir hindered the inflammatory response augment induced by sepsis, presenting as the downregulation in TNF-α, IL-1β, IL-6 (*P* < 0.05).

*** *P* < 0.001 vs. control group, **^###^** *P* < 0.001 vs. CLP group.

## Discussion

Sepsis is a syndrome of infection-induced organ dysfunction [18]. Nowadays, due to the relative difficulty of clinical diagnosis and treatment, sepsis has always maintained a high morbidity and mortality in the field of critical diseases [19]. From the available serum biomarkers for the diagnosis of sepsis, CRP, PCT, CD14, suPAR and other biomarkers showed good correlations for the diagnosis and treatment of sepsis, but their specificity and sensitivity are still not ideal for the diagnostic efficacy [20,21]. Therefore, a more in-depth study of sepsis-related biomarkers is needed to provide evidence for early clinical diagnosis and treatment of sepsis.

Circulating miRNA refers to miRNA presented in extracellular fluids, which are not affected by RNase, storage, and acid-base conditions. To date, a large amount of data have focused on circulating miRNAs as potential biomarkers for research, involving tumors, metabolic diseases, inflammatory diseases, and so on. Notably, studies have shown that miRNA can be used as a biomarker to assist in the diagnosis of sepsis [22,23]. For example, Wang et al. observed that serum miR-223 and miR-146a exhibited a significant downward trend in sepsis patients compared to patients with SIRS. Further study found that there was no difference in miR-223 changes in SIRS patients and healthy controls, which means that miR-223 and miR-146a can effectively distinguish SIRS patients from sepsis patients [24]. In the present study, it was the first time we have found that miR-378a-3p has a significantly high expression in the serum of patients with sepsis. After data analysis, we were surprised to see that the level of miR-378a-3p was correlated with several evaluation indicators of sepsis. Our subsequent analysis on ROC curve concluded that miR-378a-3p is of considerable clinical significance in the diagnosis of sepsis.

Cardiac dysfunction and myocardial damage induced by sepsis are the direct causes of the high mortality of this disease. Septic cardiac dysfunction is a reversible functional change in the inherent systolic and/or diastolic function of myocardium caused by sepsis, and early cardioprotective therapy may be beneficial for patients with sepsis. Previous studies have reported that abnormal expression of miR-378a-3p in myocardial tissue of patients with end-stage heart failure [25]. Ganesan et al. showed that high expression of miR-378a can cause K-ion channel dysfunction in cardiac stem cells and lead to cardiac hypertrophy [26]. Knezevic et al. reported that expression of miR-378a-3p increased apoptosis of cardiomyocytes by targeting IGF1R [27]. Shao et al. reported that mesenchymal stem cell-derived exosomes (MSCs-exo) may promote the repair of cardiac function by down-regulating miR-378a [28]. Clinically, the logistic regression analysis proved miR-378a-3p to be an independent influencing factor of cardiac dysfunction for sepsis patients. Our study further successfully constructed a rat model of sepsis by CLP surgery, and then the relationship between miR-378a-3p and septic cardiac dysfunction in vivo was explored. In this study, increasing miR-378a-3p expression was observed in the serum of septic rats. Both LVSP and +dp/dtmax were declined in the sepsis rat, while LEVDP and -dp/dtmax were increased, indicating that the weakening of myocardial contractility of animals. From this, we obtained that sepsis fundamentally affected the systolic and diastolic functions of the myocardium. cTnI and CK-MB are commonly used in clinical evaluation of myocardial status and are specific markers of myocardial injury [29,30]. We noticed that the levels of cTnI and CK-MB were increased in the serum of CLP group, suggesting the cardiac dysfunction of sepsis rat was accompanied by the occurrence of myocardial injury. At the same time, the experimental results also proved that the high expression of miR-378a-3p in sepsis was an adverse factor for cardiac function. Further studies showed that the hemodynamic parameters and myocardial injury indexes of the rats were significantly improved after the injection of miR-378a-3p antagomir compared with the CLP group, suggesting that down-regulation of miR-378a-3p is conducive to the development of cardiac function in a favorable direction. This conclusion is consistent with previous studies by Shao et al. [28].

The relationship between sepsis and inflammation is self-evident. The participation of miR-378a-3p in inflammatory response in nonalcoholic fatty liver disease has been demonstrated in Zhang et al. [31]. Therefore, this study also explored the inflammatory response in sepsis. Results confirmed that inhibition of miR-378a-3p expression could significantly reduce the level of TNF-α, IL-1β, IL-6 in the serum of sepsis rats, and thus improved the inflammatory response induced by sepsis. Together, the above experimental data manifested that highly expressed miR-378a-3p exacerbated sepsis by disrupting normal cardiac function and promoting inflammatory response.

## Conclusion

In summary, our study confirmed the abnormal expression of miR-378a-3p in sepsis and the potential of this miRNA as a diagnostic biomarker for sepsis. And the slience of miR-378a-3p could improve cardiac function and reduce inflammatory response in rats. These findings may provide some basis for the understandings of sepsis.

## Data Availability

To the extent reasonable, the corresponding author may provide data relevant to the study.

## Highlights

1. The expression of miR-378a-3p was elevated in the serum of sepsis patients and sepsis rat model, and the high expression of miR-378a-3p may be a potential biomarker for the diagnosis of sepsis.

2. Overexpression of miR-378a-3p promoted cardiac dysfunction and inflammatory response in sepsis rat models.

3. miR-378a-3p antagomir down-regulated the expression of miR-378a-3p and significantly alleviated cardiac dysfunction and reduced the production of inflammatory factors in sepsis rat model.
